# Supplementary material for: Generation of bright autobioluminescent bacteria by chromosomal integration of the improved lux operon ilux2
Source: Sci Rep. 2022 Nov 9;12:19039. doi: 10.1038/s41598-022-22068-5 (PMC9646698; doi:10.1038/s41598-022-22068-5)
Supplement: Supplementary file 6 — Supplementary Information 1. [file 41598_2022_22068_MOESM6_ESM.pdf]

Generation of bright autobioluminescent bacteria by chromosomal integration of the improved *lux* operon *ilux2*

Carola Gregor[1,2,3*]

[1]Max Planck Institute for Multidisciplinary Sciences, Department of NanoBiophotonics, Göttingen, Germany

[2]Institut für Nanophotonik Göttingen e.V., Department of Optical Nanoscopy, Göttingen, Germany

[3]Cluster of Excellence "Multiscale Bioimaging: from Molecular Machines to Networks of Excitable Cells" (MBExC), University of Göttingen, Göttingen, Germany

*Correspondence should be addressed to C.G. (carola.gregor@ifnano.de).

Supplementary Tables

Supplementary Table S1. Plasmid combinations used for error-prone mutagenesis. pGEX(-) was used as the vector backbone.

Plasmid for error-prone mutagenesis				Plasmid containing the remaining lux genes			
Insert	Promoter	Origin of replication	Resistance	Insert	Promoter	Origin of replication	Resistance
iluxAB	lac	p15Aori	ampicillin	*iluxCDEfrp*	tac	ColE1	kanamycin
iluxCDE	lac	p15Aori	ampicillin	*iluxABfrp*	tac	ColE1	kanamycin
ilux frp	tac	p15Aori	ampicillin	*iluxCDABE*	tac	ColE1	kanamycin

Supplementary Table S2. Mutations identified during the screening of *luxAB* in addition to the mutations in *ilux*.

Gene	Mutations
iluxXA	L24M, I76V, Q86H, M139L, K283R, N289S, I298T, Y360-
iluxXB	T28S, Q95H, D112G, K193R, K238N, Y276F, M306T, V311A, D317A, H323L

Supplementary Table S3. Mutations contained in *ilux2* in addition to the mutations in *ilux*.

Gene	Mutations
ilux2C	T10N, N27T, N45T, I47T, E54D, D74N, S77P, L132I, D135G, T216I, Y248N, K345N, V348A, K414E, T445K
ilux2D	N3K, I90T, K106N, N108S, M112V, N165K, D168V, K234R, V251A, D274N, E300V

Supplementary Table S4. Primer sequences.

Name	Sequence (5'→3')
KanR SpeI fwd	GTTGATACTAGTATGAGCCATATTCAACGG
KanR AscI rev	GTTGATGGCGCGCCTTAGAAAAACTCATCGAG
pGEX(-) SpeI rev	GTTGCAACTAGTACTCTTCCTTTTTCAATATTATTG
pGEX(-) AscI fwd	GTTGATGGCGCGCCCTGTCAGACCAAGTTTACTCA
pGEX(-) +lac BamHI rev	TCCGGGGATCCTGTTTCCTGTGTGAAATTGTTATCCGCTCACAATTCCACACCAACATACGAGCCGGAAGCATAAAGTGTAAACAGCTCATTTCAGAAT
pGEX(-) BamHI fwd	GGATAACAATTTCACACAGGAAACAGGATCC
p15Aori BglII fwd	ATCTTCAGATCTTTGAGATCGTTTTGGTCT
p15Aori AvrII rev	TTGATCCCTAGGTTTCCATAGGCTCCGCCC
pGEX(-) BglII rev	AACTAGAGATCTGAAGATCCTTTGATCTTTTCTAC
pGEX(-) AvrII fwd	GTTGCACCTAGGAACGCCAGCAACGCGGCC
pGEX(-) AscI tac fwd	TTGATAGGCGCGCCTTGACAATTAATCATCGGCTCGTATAA
pGEX(-) AscI tac rev	GTTACTGGCGCGCCCAGCTCATTTCAGAATATTTGCCAG
attTn7 fwd	GATGCTGGTGGCGAAGCTGT
luxC EcoRI rev	GTTGATGAATTCACTTTTTACCTATTATGGGACAAATACAAGGAAC
frp SalI fwd	GTAACTGTCGACCTAAGGAGAAAGAAATGGTGAAGATACAG
attTn7 rev	GATGACGGTTTGTCACATGGA

Supplementary Figures

>*ilux2C*

ATGACTAAAAAAATTTCATTCATTATTAATGGCCAGGTAGAAATCTTTCCCGAAAGCGAT
GATTTAGTGCAATCCATCACTTTTGGTGATAATAGTGTTTACCTGCCAATATTGAACGAC
TCTCATGTAAAAACCATTACTGATTGTAATGGAAATAACGACTTACGGTTGCATGACATT
GTCAATTTTCTCTATACGGTAGGGCAAAGATGGAAAAATAATGAATACCCAAGACGCAG
GACATACATTCGTGACTTAAAAAAATATATGGGATATTCAGAAGAAATGGCTAAGCTAGA
GGCCAATTGGATATCTATGATATTATGTTCTAAAGGCGGCCTTTATGATGTTGTAGAAAA
TGAACTTGGTTCTCGCCATATCATGGATGAATGGATACCTCAGGGTGAAAGTTATGTTC
GGGCTTTTCCGAAAGGTAAATCCGTACATCTGTTGGCAGGTAATGTTCCATTATCTGGG
ATCATGTCTATATTACGTGCAATTTTAACTAAGAATCAGTGTATTATAAAAACATCGTCAA
CCGATCCTTTTACCGCTAATGCATTAGCTTTAAGTTTTATTGATGTAGACCCTAATCATCC
GATAACGCGCTCTTTATCTGTTATATATTGGCCCCACCAAGGTGATATATCACTCGCAAA
AGAAATTATGCGACATGCGGATGTTATTGTCGCATGGGGAGGGCCAGATGCGATTAATT
GGGCGGTAGAGCATGCGCCATCTAATGCTGATGTGATTAAATTTGGTCCTAAAAAGAGC
CTTTGCATTATCGATAATCCTGTTGATCTGACGTCTGCAGCGACAGGTGCGGCTCATGA
TGTTTGTTTTTACGATCAGCGAGCTTGCTTTTCTGCCCAAAACATATATTATATGGGAAAT
CATTATGAGGAATTTAAGTTGGCATTGATAGAAAAACTTAATCTATATGCGCATATATTAC
CGAATGCAAAAAAAGATTTTGATGAAAAGGCGGCCTATTCTTTAGTTCAAAAAGAGAGCT
TGTTTGCCGGATTAAATGTAGAGGCGGATATTCATCAACGTTGGACGATTATTGAGTCA
GATGCAGGTGTGGAATTTAATCAACCACTTGGCAGATGTGTGTACCTTCATCACGTCGA
TAATATTGAGCAAATATTGCCTTATGTTCAAAAAAATAAGACGCAAACCATATCTATTTTT
CCTTGGGAGTCATCATTTAAATATCGAGATGCGTTAGCATTAGAAGGTGCGGAAAGGAT
TGTAGAAGCAGGAATGAATAACATATTTCGAGTTGGTGGATCTCATGACGGAATGAGAC
CGTTGCAACGATTAGTGAAGTATATTTCTCATGAAAGGCCATCTAACTATACGGCTAAGG
ATGTTGCGGTTGAAATAGAACAGACTCGTTTCCTGGAAGAAGACAAGTTCCTTGTATTTG
TCCCATAA

>*ilux2D*

ATGGAAAAAGAATCAAAATATAAAACCATCGACCACGTTATTTGTGTTGAAGGAAATAAA
AAAATTCATGTTTGGGAAACGCTGCCAGAAGAAAACAGCCCAAAGAGAAAGAATGCCAT
TATTATTGCGTCTGGTTTTGCCCGCAGGATGGATCATTTTGCTGGACTGGCGGAATATTT
ATCGCGGAATGGGTTTCATGTGATCCGCTATGATTCGCTTCACCACGTTGGATTGAGTT
CAGGGACAATTGATGAATTTACAATGTCTACAGGAAAGCAGAGCTTGTTAGCAGTGGTT
GATTGGTTAACTACACGAAATATAAGTAACTTCGGTGTGTTGGCTTCAAGCTTATCTGCG
CGGATAGCTTATGCAAGCCTATCTGAAATCAATGCTTCGTTTTTAATCACCGCAGTCGGT

GTTGTTAACTTAAGATATTCTCTTGAAAGAGCTTTAGGGTTTGATTATCTCAGTCTTCCCA
TTAATGAATTGCCGAAAAATCTAGTTTTTGAAGGCCATAAATTGGGTGCTGAAGTCTTCG
CGAGAGATTGTCTTGATTTTGGTTGGGAAGACTTAGCTTCTACAATTAATAACATGATGT
ATCTTGATATACCGTTTATTGCTTTTACCGCAAATAACGACAATTGGGTCAAGCAAGATG
AAGTTATCACATTGTTATCAAATATTCGAAGTAATCGATGTAGGATATATTCTTTGTTAGG
AAGTTCGCATGACTTGAGTGAAAATTTAGTGGCCCTGCGCAATTTTTATCAATCGGTTAC
GAAAGCCGCTATCGCGATGGATAATGATCATCTGGATATTAATGTTGATATTACTGAACC
GTCATTTGAACATTTAACTATTGCGACAGTCAATGAACGCCGAATGAGAATTGAGATTGT
AAATCAAGCAATTTCTCTGTCTTAA

>*ilux2A*

ATGAAATTTGGAAACTTTTTACTTACATACCAACCTCCCCAATTTTCTCAAACAGAGGTAA
TGGAACGTTTGGTTAAATTAGGTCGCATCTCTGAGGAGTGTGGTTTTGATACCGTATGG
TTACTGGAGCATCATTTCACGGAGTTTGGTCTACTTGGTAACCCTTATGTCGCTGCTGCA
TATTTACTTGGCGCGACTAAAAAATTGAATGTAGGAACCGCCGCTATTGTTCTTCCCACA
GCCCATCCAGTACGCCAACTTGAAGATGTGAATTTATTGGATCAAATGTCAAAAGGACG
ATTTCGGTTTGGTATTTGCCGAGGGCTTTACAACAAGGACTTTCGCGTATTCGGCGCGG
ATATGAATAACAGTCGCGCCTTAGCGGAATGCTGGTACGGGCTGATAAAGAATGGCATG
ACAGAGGGATATATGGAAGCTGATAATGAACATATCAAGTTCCATAAGGTAAAAGTAAAC
CCCGCGGCGTATAGCAGAGGTGGCGCACCGGTTTATGTGGTGGCTGAATCAGCTGCGA
CGACTGAGTGGGCAGCTCAATTTGGCCTACCGATGATATTAAGTTGGATTATAAATACTA
ACGAAAAGAAAGCACAACTTGAGCTTTATAATGAGGTGGCTCAAGAATATGGGCACGAT
ATTCATAATATCGACCATTGCTTATCATATATAACATCTGTAGATCATGACTCAATTAAAG
CGAAAGAGATTTGCCGGAAATTTCTGGGGCATTGGTATGATTCTTATGTGAATGCTACG
ACTATTTTTGATGATTCAGACCAAACAAGAGGTTATGATTTCAATAAAGGGCAGTGGCGT
GACTTTGTATTAAAAGGACATAAAGATACTAATCGCCGTATTGATTACAGTTACGAAATC
AATCCCGTGGGAACGCCGCAGGAATGTATTGACATAATTCAAAAAGACATTGATGCTAC
AGGAATATCAAATATTTGTTGTGGATTTGAAGCTAATGGAACAGTAGACGAAATTATTGC
TTCCATGAAGCTCTTCCAGTCTGATGTCATGCCATTTCTTAAAGAAAAACAACGTTCGCT
ATTATATTAG

>*ilux2B*

ATGAAATTTGGATTGTTCTTCCTTAACTTCATCAATCCGACAACTGTTCAAGAACAAAGTA
TAGTTCGCATGCAGGAAATAACGGAGTATGTTGATAAGTTGAATTTTGAACAGATTTTAG
TGTATGAAAATCATTTTTCAGATAATGGTGTTGTCGGCGCTCCTCTGACTGTTTCTGGTT
TTCTGCTCGGTTTAACAGAGAAAATTAAAATTGGTTCATTAAATCACATCATTACAACTCA
TCATCCTGTCCGCATAGCGGAGGAAGCTTGCTTATTGGATCAGTTAAGTGAAGGGAGAT

TTATTTTAGGGTTTAGTGATTGCGAAAAAAAAGATGAAATGCATTTTTTTAATCGCCCGG
CTGAATATCAACAGCAACTATTTGAAGAGTGTTATGAAATCATTAACGATGCTTTAACAAC
AGGCTATTGTAATCCAGATAACGATTTTTATAGCTTCCCTAAAATATCTGTAAATCCCCAT
GCTTATACGCCAGGCGGACCTCGGAAATATGTAACAGCAACCAGTCATCATATTGTTGA
GTGGGCGGCCAAAAAAGGTATTCCTCTCATCTTTAAGTGGGATGATTCTAATGATGTTAG
ATATGAATATGCTGAAAGATATAAAGCCGTCGCGGATAAATATGACGTTGACCTATCAGA
GATAGACCATCAGTTAATGATATTAGTTAACTATAACGAAGATAGTAATAAAGCTAAACAA
GAGACGCGTGCATTTATTAGTGATTATGTTCTTGAAATGCACCCTAATGAAGATTTCGAA
AATAAACTTGAAGAAATAATTGCAGAAAACGCTGTCGGAAATTATACGGAGTGTATAACT
GCGGCTAAATTGGCAATTGAAAAGTGTGGTGCGAAAAGTGTATTGCTGTCCTTTGAACC
AATGAATGATTTGATGAGCCAAAAAAATGTAATCAATATTGTTGATGATAATATTAAGAAG
TACCACATGGAATATACCTAA

>*ilux2E*

ATGACTTCATATGTTGATAAACAAGAAATTACAGCAAGCTCAGAAATTGATGATTTGATTT
TTTCGAGCGATCCATTAGTGTGGTCTTACGACGAGCAGGAAAAAATCAGAAAGAAACTT
GTGCTTGATGCATTTCGTAATCATTATAAACATTGTCGAGAATATCGTCACTACTGTCAG
GCACACAAAGTAGATGACAATATTACGGAAATTGATGACATACCTGTATTCCCAACATCG
GTTTTTAAGTTTACTCGCTTATTAACTTCTCAGGAAAACGAGATTGAAAGTTGGTTTACCA
GTAGCGGCACGAATGGTTTAAAAAGTCAGGTGGCGCGTGACAGATTAAGTATTGAGAGA
CTCTTAGGCTCTGTGAGTTATGGCATGAAATATGTTGGTAGTTGGTTTGATCATCAAATA
GAATTAGTCAATTTGGGACCAGATAGATTTAATGCTCATAATATTTGGTTTAAATATGTTA
TGAGTTTGGTGGAATTGTTATATCCTACGACATTTACCGTAACAGAAGAACGAATAGATT
TTGTTAAAACATTGAATAGTCTTGAACGAATAAAAAATCAAGGGAAAGATCTTTGTCTTAT
TGGTTCGCCATACTTTATTTATTTACTCTGCCATTATATGAAAGATAAAAAAATCTCATTTT
CTGGAGATAAAAGCCTTTATATCATAACCGGAGGCGGCTGGAAAAGTTACGAAAAAGAA
TCTCTGAAACGTGATGATTTCAATCATCTTTTATTTGATACTTTCAATCTCAGTGATATTA
GTCAGATCCGAGATATATTTAATCAAGTTGAACTCAACACTTGTTTCTTTGAGGATGAAAT
GCAGCGTAAACATGTTCCGCCGTGGGTATATGCGCGAGCGCTTGATCCTGAAACGTTG
AAACCTGTACCTGATGGAACGCCGGGGTTGATGAGTTATATGGATGCGTCAGCAACCA
GTTATCCAGCATTTATTGTTACCGATGATGTCGGGATAATTAGCAGAGAATATGGTAAGT
ATCCCGGCGTGCTCGTTGAAATTTTACGTCGCGTCAATACGAGGACGCAGAAAGGGTG
TGCTTTAAGCTTAACCGAAGCGTTTGATAGTTGA

>*ilux2frp*

ATGGTGAAGATACAGCCCATCCCCACAACTAGCCAGGGCAGCCTTTTTATAATGAATAG
CACCATAGAGACAATCCTGGGCCATAGATCCATTAGGAAGTTCACATCTGAACCTATTG

CTAGTGAGCAGCTGCAAACGATTCTTCAGTCTGGGCTCGCTGCTTCAAGCTCATCCATG
CTGCAGGTTGTGAGTATAATTCGGGTTACAGACACGGAAAAGAGAAAATTGCTCGCTCA
ATATGCCGGCAACCAGACGTATGTGGAATCCGCTGCTGAGTTCCTGGTCTTTTGTATAG
ACTACCAGCGACACGCTACTATCAACCCCGATGTCCAAGCTGACTTTACCGAGCTGACC
CTGATTGGTGCAGTGGATTCCGGCATAATGGCCCAGAATTGCCTCCTGGCAGCAGAAT
CAATGGGTCTTGGCGGAGTCTATATCGGAGGACTTCGGAACTCAGCTGCCCAAGTGGA
TGAGTTGCTCGGACTGCCCAAGAACACAGCTATCCTCTTCGGAATGTGCTTGGGGCAC
CCCGATCAGAGCCCTGAGACAAAGCCTAGACTGCCCGCTCATGTGATCGTGCACGAGA
ACCAATATCAAGCTCTGAACATTGACGACGTACAGGCGTATGACAAAACATTGCAGGAG
TATTATGCCAGCAGAACCAGCAACCAGAAGCAGAGTGTCTGGTCCCAGGAAACTGCAG
GCAAGCTGGCCGGAGAATCCCTCCCACACATCCTGCCATACCTGAACTCCAAGGGCCT
TGCCAGAAGGTAA

Figure S1. Nucleotide sequences of the *ilux2* genes. The sequences of *ilux2A*, *ilux2B* and *ilux2frp* are identical to *iluxA*, *iluxB* and *iluxfrp*, respectively. The sequence of *ilux2E* is the same as in the wild-type *lux* operon.

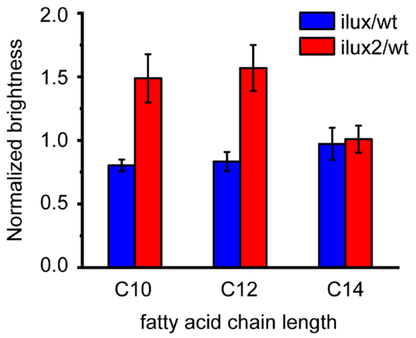

Figure S2. Comparison of aldehyde production of LuxCDE wt, iLuxCDE and iLux2CDE from saturated fatty acids of different chain length. Top10 cells expressing *luxCDE wt*, *iluxCDE* or *ilux2CDE* from pGEX(-) Kan were incubated in LB medium containing 300 µM decanoic, dodecanoic or tetradecanoic acid for 5 min. Subsequently, Top10 cells expressing *iluxABfrp* from pGEX(-) Kan were added to detect the aldehyde produced by LuxCDE by bioluminescence. Imaging was performed at room temperature. For each fatty acid, the bioluminescence signal was normalized to LuxCDE wt. Error bars represent SD of 4 different clones.

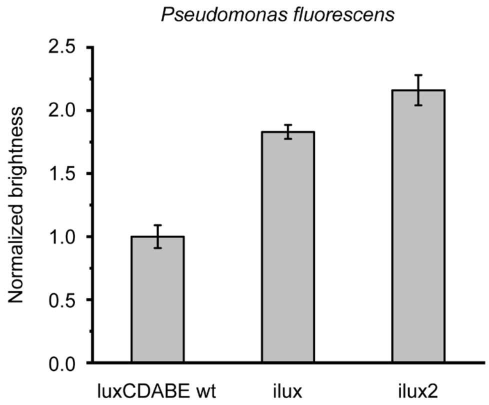

Figure S3. Comparison of *luxCDABE wt*, *ilux* and *ilux2* in *Pseudomonas fluorescens*. The indicated *lux* operons were expressed in *P. fluorescens* from the vector pJOE7771.1 at 30 °C. The bioluminescence signal was normalized to the *luxCDABE wt* operon. Error bars represent SD of 5 different clones.

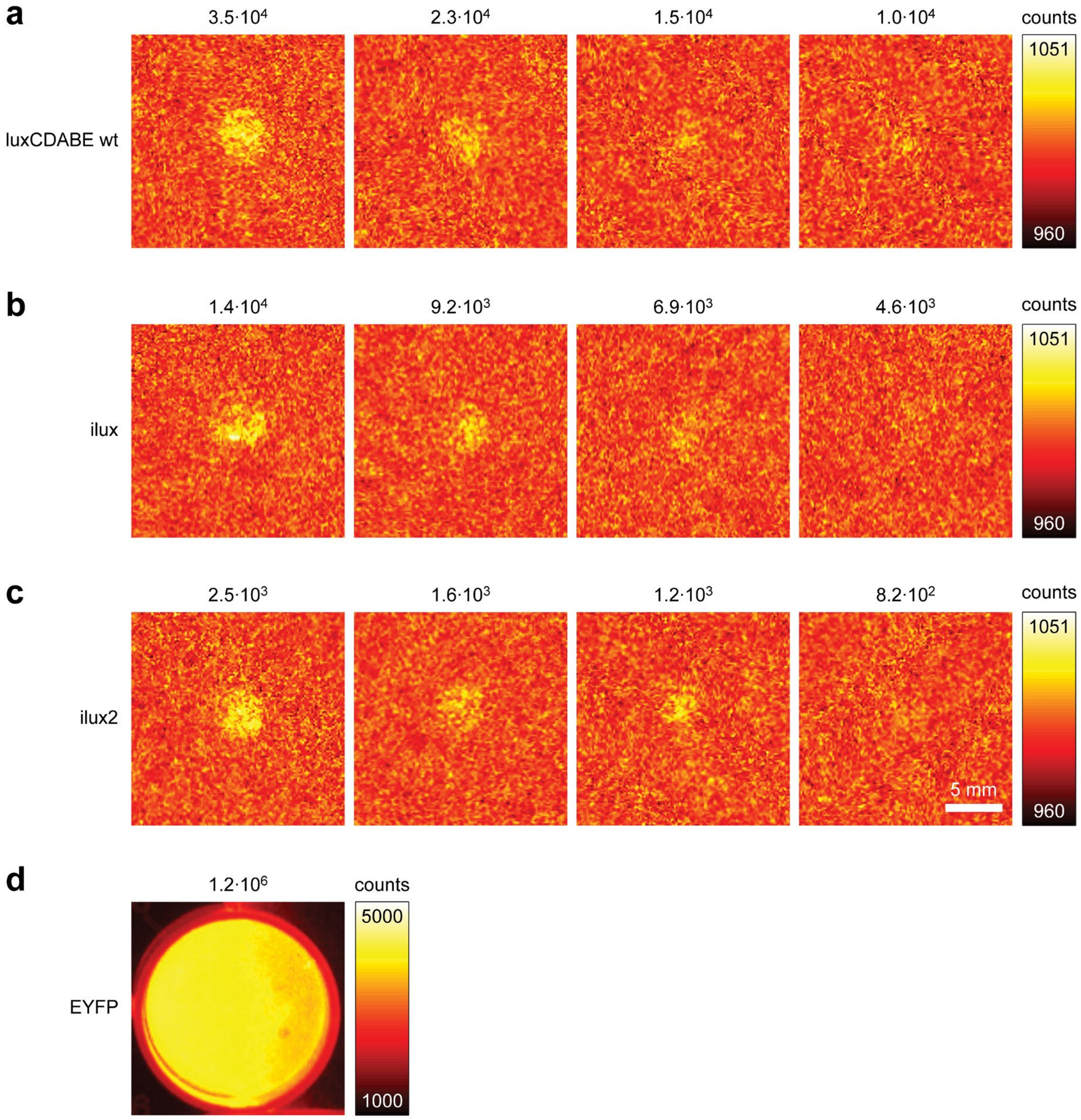

Figure S4. Detection of Top10 strains chromosomally labeled with (**a**) *luxCDABE wt*, (**b**) *ilux*, (**c**) *ilux2* or (**d**) EYFP on mashed potatoes. The indicated numbers of colony-forming units were applied in 1 µl PBS onto mashed potatoes in 24-well plates. Imaging was performed at room temperature. Bioluminescence from the *luxCDABE wt*, *ilux* and *ilux2* strains was imaged with an exposure time of 10 min. Fluorescence of the EYFP strain was excited at 520 nm and imaged with an exposure time of 1 s.

Supplementary Movies

Movie S1 (separate file). *E. coli* Top10 strain chromosomally labeled with *ilux2* grown on cucumber. A slice of cucumber was inoculated with $1{\cdot}10^6$ colony-forming units (cfu). Bioluminescence images were taken at room temperature with an Amersham Imager AI 600 RGB using an exposure time of 1 min per image.

Movie S2 (separate file). *E. coli* Top10 strain chromosomally labeled with *ilux2* grown on potato. A slice of raw potato was inoculated with $1{\cdot}10^6$ cfu. Bioluminescence images were taken at room temperature with an Amersham Imager AI 600 RGB using an exposure time of 1 min per image.

Movie S3 (separate file). *E. coli* Top10 strain chromosomally labeled with *ilux2* grown on mashed potatoes. A dish filled with mashed potatoes was inoculated with $5{\cdot}10^5$ cfu. Bioluminescence images were taken at room temperature with an Amersham Imager AI 600 RGB using an exposure time of 5 s per image.

Movie S4 (separate file). *E. coli* Top10 strain chromosomally labeled with *ilux2* grown on egg yolk. A dish filled with raw egg yolk was inoculated with $5{\cdot}10^5$ cfu. Bioluminescence images were taken at room temperature with an Amersham Imager AI 600 RGB using an exposure time of 5 s per image.

Movie S5 (separate file). *E. coli* Top10 strain chromosomally labeled with *ilux2* grown in milk. A dish filled with UHT milk was inoculated with $3{\cdot}10^6$ cfu. Bioluminescence images were taken at room temperature with an Amersham Imager AI 600 RGB using an exposure time of 1 min per image.
